# Toothbrushing behavior over time: A correlational analysis of repeatedly assessed brushing performance

**DOI:** 10.1371/journal.pone.0296724

**Published:** 2024-12-19

**Authors:** Ulrike Weik, Thorben Sämann, Zdenka Eidenhardt, Sadhvi Shankar Subramanian, Bernd Wöstmann, Jutta Margraf-Stiksrud, Renate Deinzer

**Affiliations:** 1 Institute of Medical Psychology, Justus-Liebig-University Giessen, Giessen, Germany; 2 Department of Prosthodontics, Dental Clinic, Justus-Liebig-University Giessen, Giessen, Germany; 3 Philipps-University Marburg, Marburg, Germany; Public Library of Science, UNITED STATES OF AMERICA

## Abstract

Effective toothbrushing is important for maintaining good oral health. Nonetheless, epidemiological data suggest that individuals often encounter challenges in achieving oral cleanliness when brushing their teeth. Various cross-sectional studies have observed brushing behavior of individuals and found some widespread behavioral deficits including neglect of inner surfaces and inconsistent brushing movements. However, longitudinal studies exploring the consistency of these deficits over time are currently lacking. Therefore, this study investigated the intra-individual stability of toothbrushing performance by repeated observation of 105 students during two brushing appointments (T1/T2) two weeks apart. One-half of the participants (n = 52) were instructed to brush to the best of their ability, while the other half were directed to brush as usual (n = 53). Calibrated observers analyzed brushing duration, brushing movements (horizontal, circular, vertical), brushing time on tooth surfaces (outer, inner, occlusal), and distribution of time across sextants. Correlational analyses revealed a high intra-individual stability of all parameters in both groups. Correlation coefficients varied between r = 0.72 (horizontal movements at outer surfaces) and r = 0.93 (total tooth contact time). All correlations were statistically significant (p < 0.001). The results indicated that specific individual toothbrushing patterns were developed by the participants. It is important that preventive measures take into account the challenge of changing such established habits. Healthcare professionals and patients should recognize this challenge when addressing oral hygiene deficiencies.

## Introduction

Thorough oral hygiene is a preventive self-care behavior for maintaining oral health [1–4]. However, the high prevalence of plaque-associated periodontal disease [5–7] suggests that the brushing behavior of individuals is ineffective at ensuring oral cleanliness. Several studies have consistently shown residual plaque immediately after toothbrushing, particularly on gingival margin sections and inner teeth surfaces [8–18]. Video-based observational studies indicate that this inadequate plaque removal might be a result of suboptimal oral hygiene skills [13, 15, 18–22]. In these studies, brushing performance was assessed by the analysis of brushing time and distribution across teeth surfaces and sextants as well as brushing movements. The results revealed that many study participants did not brush at least one sextant and neglected the inner teeth surfaces; in some cases, the latter were omitted completely. Regarding brushing techniques, study participants frequently showed only horizontal brushing movements as opposed to applying more elaborate circular or vertical movements [18–22].

These observational studies were of cross-sectional nature and provided limited insight into whether the toothbrushing behavior observed once is maintained over time. Survey studies indicate that toothbrushing is generally performed regularly as a part of daily routine [23–25], which indicates that the process has a habitual nature. Habits are established as a result of a repeated and patterned sequence of behavior that becomes routine and automatic over time [26–28]. Children learn to brush their teeth at a very early age with adult support and supervision [29], and over time a toothbrushing habit is formed that is automatic and performed more-or-less subconsciously [26, 28]. This suggests that once the habit is established, it tends to remain unchanged over time. However, there is a paucity of empirical work on habit formation most of which focuses on specific aspects such as the cues that trigger the brushing behavior [30], the frequency of brushing, and the sequence of actions performed [26].

In terms of toothbrushing performance itself, there is some data on the stability of specific aspects of the performance such as brushing duration or the brushing force. For example, it has been shown that children or students show little variation in brushing time, brushing force, or other brushing patterns, such as the surfaces of the teeth brushed or the brushing technique used [31–33]. However, these studies are somewhat dated or based on only a small subsample of study participants. Furthermore, most involved only a few aspects of the toothbrushing behavior such as brushing duration or brushing force. Thus, it still remains uncertain, which further parts of the brushing performance are shown in a stable form or vary randomly from brushing to brushing event. This knowledge is required to alter aspects of brushing behavior and improve its effectiveness.

The aim of the present study was to examine the degree to which behavioral aspects of toothbrushing performance remain stable over time or vary between brushing events. The hypothesis tested was that there is a high degree of concordance between two toothbrushing events assessed two weeks apart. This hypothesis is tested under two conditions: When study participants are instructed to perform to the best of their ability and as usual.

## Methods

The study included two study objectives and was registered with the German Clinical Trials Register (www.drks.de; ID: DRKS00017812; 2019). The present analysis focuses on the second objective, which involved assessing the stability of the behavioral parameters observed at two time points (T1 and T2) two weeks apart. The first objective was to analyze the differences in brushing performance and the subjective and objective achieved oral cleanliness under two different brushing instructions. The respective analyses considered the data of T2 only, and were published previously [34].

### Ethics

The study was conducted according to the principles of the Declaration of Helsinki. The Ethics Committee of the Department of Medicine at Justus-Liebig-University in Giessen, Germany approved the study protocol (file no 254/18; 2019/01/23). Study participants were recruited from April 5, 2019 through July 17, 2019. The study participants received detailed information before the start of the examination and all participants provided written consent.

### Study sample

The study participants were university students from Giessen (Hesse, Germany), aged 18–35 years, who predominantly (at least 2/3 of all brushing events) brushed their teeth using a manual toothbrush. Participants were recruited through the ‘internal email distribution list of the university, which includes almost all students of the university, as well as through advertisements in a regional online magazine. Students were excluded if they: (1) were studying dentistry or human medicine, (2) had fixed orthodontic appliances, removable prostheses/dentures, dental jewelry, or oral piercings, (3) had a physical impairment that affected their oral hygiene behavior, (4) used antibiotics within the three months prior to study entry, (5) had dental prophylaxis within the four months prior to the study entry, or (6) were pregnant. The sample size was calculated using the free available power analysis program G*Power [35] based on the first study objective (comparison of brushing to the best of one’s ability vs. as usual) and resulted in a minimal sample size of n = 102 (see [34]). This sample size allowed for the detection of correlations of ρ > 0.72 with alpha = 0.05 and a power of 1 - β = 0.80.

### Procedures

The study was conducted at the Institute of Medical Psychology of the Justus-Liebig-University in Giessen, Germany. The procedures followed those previously described [34] and provided in S1 File. Briefly, eligible students were informed of the study details and scheduled for two brushing sessions two weeks apart. The procedures during the two appointments (T1 and T2) were the same. While participants brushed their teeth at both T1 and T2, clinical data were not assessed on both appointments. Disclosing of teeth and assessment of dental plaque took place only at T2. To ensure both appointments were as similar as possible and to prevent visible plaque staining at T1 from influencing brushing behavior at T2, a sham staining (using water faked as a fluorescent solution) and a simulated plaque assessment were conducted at T1. With regard to the first study objective (not focused here), study participants were randomly assigned to two brushing conditions (brushing to the best of one’s abilities vs. brushing as usual). At both appointments, the study participants brushed their teeth according to their assigned brushing instructions. The data assessors were maintained between the two appointments and blinded to the respective brushing condition. Any interaction with the study participants was conducted in a fully standardized manner. For both brushing sessions, the study participants were placed in front of a mobile washbasin and a computer tablet with a front camera mounted on a tripod. The tablet served as a mirror for the participants as well as a device for recording toothbrushing performance. In addition, toothbrushing was recorded by two side cameras mounted on the walls in case the tablet camera did not fully capture the brushing event. The participants were provided with a standard manual toothbrush (Elmex InterX short brush-head, medium; CP GABA, Hamburg, Germany) and toothpaste (Elmex; CP GABA). Dental floss (waxed and unwaxed; Elmex; CP GABA), super floss (Meridol Special-Floss; CP GABA), and interdental brushes (Elmex interdental brush sizes 2 and 4; CP GABA) were provided on a table beneath the basin. After receiving their brushing instructions, the participants were asked to begin brushing their teeth and their brushing performance was recorded.

### Observed behavioral parameters

The video-based analysis of the brushing behavioral parameters was conducted according to the procedure described in previous studies [17, 21]. A detailed description is provided in the supplemental material. The videos were analyzed by independent calibrated observers using observational software (Interact 18; Mangold International; Arnsdorf, Germany). Videos from a previous study were used for calibration. The criterion for a successful calibration was an intra-class correlation (ICC) ≥ 0.90 on five consecutively analyzed videos for each of the observed behavioral parameters. To ensure the reliability of the video analysis, an additional 10 videos of the study participants were analyzed in duplicate by two independent observers. These double codings returned a high agreement between the respective independent observers (ICC ≥ 0.90 for all observed behavioral parameters). The behavioral parameters included were: a)total tooth contact time (tct; length of time (seconds) that the brush touched the teeth), b) tct and proportional distribution of tct on the tooth surface (occlusal, outer and inner teeth surfaces), c) tct of circular or horizontal brushing movements on outer surfaces and vertical and horizontal movements on inner surfaces, and d) overall quality index for the distribution of tct across sextants and tooth surfaces (QIT-S [19]; this index represents a rank-scaled measure describing the extent to which the sextants were brushed on the outer and inner surfaces).

### Statistical analysis

The present data analysis was conducted to test the hypothesis that the described toothbrushing parameters observed at T1 and T2 are highly correlated regardless of whether the study participants brushed to the best of their ability or as usual. According to Gilford [36], correlational coefficients between 0.70 and 0.90 indicate a high correlation, whereas correlations above 0.90 describe a very high correlation and a reliable relationship.

A re-test correlation of r > 0.70 is sufficiently high to render brushing behavior stable over time. In addition to the product-moment correlational coefficient (Pearson), rank correlation coefficients (Spearman) were calculated to account for potential outlier values. According to the research hypothesis, the following hypothesis pair was independently statically tested for each of the behavioral parameters under observation: H_0_: ρ_T1/T2_ ≤ 0.5 and H_1_: ρ_T1/T2_ > 0.5. The respective means, SDs, and effect sizes with 95% confidence intervals of the differences of the means at T1 and T2 were reported. Effect sizes were calculated for correlational data according to Dunlap et al. [37]. Cohen [38] stated that effect sizes of d ≥|.2|, |.5|, and |.8| were considered small, medium, and large, respectively. The relationships between T1 and T2 of the categorical data assessed by the QIT-S index were analyzed by chi^2^-tests. All statistical analyses were conducted with the statistical software package SPSS (IBM SPSS Statistics for Windows, Version 28, IBM, Armonk, New York, USA).

## Results

Of the 106 study participants who finished the study, one person in the best-brusher group was excluded from the analyses due to an unusual brushing behavior (tooth contact time exceeded 15 minutes and deviated from the mean value by more than four SD). The demographic and clinical data of the study sample are provided in Table 1.

**Table 1:** Characteristics of the study sample.

	Brushing to the best of one’s ability (n = 52)		Brushing as usual (n = 53)	
	M (SD)			
	n/n			
Sex (f/m/x)	42/9/1		45/8/0	
Age (years)	22.77 (2.5)		23.49 (3.0)	
DMFT-index	2.77 (3.4)		3.74 (3.6)	
	*T1*	*T2*	*T1*	*T2*
PBI mean	0.73 (0.4)	0.72 (0.4)	0.64 (0.4)	0.66 (0.4)
PBI % bleeding full-mouth	43.41 (17.6)	43.22 (18.7)	38.72 (19.0)	41.02 (20.1)

DMFT: decayed, missed, filled teeth; PBI: papillary bleeding index; T1: first brushing session; T2: second brushing session

The descriptive data and results of the correlational analyses of the observed brushing parameters for each brushing group are shown in Tables 2 and 3. Scatterplots for each behavioral parameter are provided in S1 File. The descriptive analyses of the mean differences between T1 and T2 revealed small or spurious effects according to the nomenclature of Cohen (all d <|0.24|) [38]. Under both brushing instructions, the correlation coefficients were similar in magnitude and varied between r = 0.72 and r = 0.93. Total brushing time showed the greatest correlations in both groups (r = 0.90 and r = 0.93). Correlations for the absolute time values of the time spent at the tooth surfaces (occlusal, inner and outer surfaces) were r ≥ 0.83 in both groups. Similar values were observed for the percentage of time used for brushing the respective surfaces (all r ≥ 0.77). Furthermore, brushing movements were similar for the two groups, with correlations varying from r = 0.72 to r = 0.89. The lowest correlations were observed for the time by which horizontal brushing movements were observed on the outer surfaces (r = 0.72 and r = 0.73). All reported correlational coefficients were statistically significant (p < 0.001).

**Table 2:** Results of behavioral parameters (brushing to the best of one’s ability) at T1 and T2 (n = 52).

	T1		T2										
*Toothbrushing parameters*	*M*	*SD*	*M*	*SD*	*d*	[CI 95%]		*r* *	[CI 95%]		*rho* *	[CI 95%]	
Tooth contact time (tct; s)	234.06	90.15	260.02	118.73	-0.21	-0.34	-0.08	0.90	0.84	0.94	0.85	0.74	0.91
Occlusal surfaces (s)	89.11	48.01	100.23	50.09	-0.23	-0.39	-0.06	0.84	0.74	0.91	0.77	0.63	0.87
Outer surfaces (s)	106.35	48.10	111.54	54.09	-0.10	-0.23	0.04	0.89	0.81	0.93	0.84	0.73	0.91
Inner surfaces (s)	38.61	32.64	48.25	47.10	-0.21	-0.38	-0.04	0.83	0.72	0.90	0.84	0.74	0.91
Occlusal surfaces (%tct)	37.96	14.36	39.07	12.89	-0.08	-0.25	0.08	0.83	0.71	0.90	0.79	0.65	0.87
Outer surfaces (%tct)	46.51	12.59	44.41	13.84	0.16	-0.02	0.34	0.80	0.67	0.88	0.81	0.68	0.89
Inner surfaces (%tct)	15.54	10.78	16.51	11.49	-0.09	-0.28	0.10	0.77	0.63	0.86	0.78	0.64	0.87
Outer surfaces													
Horizontal movements (s)	31.80	31.81	34.10	39.91	-0.06	-0.27	0.15	0.72	0.55	0.83	0.80	0.66	0.88
Circular movements (s)	68.02	51.14	69.05	51.09	-0.02	-0.16	0.12	0.87	0.79	0.92	0.82	0.69	0.89
Inner surfaces													
Horizontal movements (s)	18.32	18.78	22.66	25.41	-0.18	-0.36	0.00	0.80	0.67	0.88	0.80	0.67	0.88
Vertical movements	15.51	23.67	19.14	33.37	-0.11	-0.28	0.06	0.81	0.70	0.89	0.67	0.49	0.80

T1: first brushing session; T2: second brushing session; tct: tooth contact time; s: seconds; d: effect size Cohen’s d for differences of means; CI 95%: 95% confidence interval

*all p < 0.001.

**Table 3:** Results of behavioral parameters (brushing as usual) at T1 and T2 (n = 53).

	T1		T2										
*Toothbrushing parameters*	*M*	*SD*	*M*	*SD*	*d*	[CI 95%]		r*	[CI 95%]		rho*	*[CI 95%]*	
Tooth contact time (tct; s)	195.85	81.90	209.38	94.57	-0.14	-0.25	-0.03	0.93	0.88	0.96	0.89	0.82	0.94
Occlusal surfaces (s)	83.47	46.26	89.90	50.84	-0.13	-0.25	-0.01	0.92	0.86	0.95	0.93	0.88	0.96
Outer surfaces (s)	84.26	37.98	85.33	41.02	-0.03	-0.13	0.08	0.92	0.87	0.96	0.89	0.81	0.93
Inner surfaces (s)	28.12	26.15	34.15	32.72	-0.19	-0.35	-0.04	0.85	0.75	0.91	0.89	0.82	0.94
Occlusal surfaces (%tct)	42.16	15.44	42.82	15.20	-0.04	-0.17	0.08	0.90	0.84	0.94	0.92	0.87	0.96
Outer surfaces (%tct)	44.47	13.27	42.05	12.33	0.19	0.05	0.32	0.89	0.81	0.93	0.87	0.78	0.92
Inner surfaces (%tct)	13.37	10.53	15.13	10.64	-0.17	-0.31	-0.02	0.87	0.78	0.92	0.87	0.79	0.93
Outer surfaces													
Horizontal movements (s)	24.69	23.73	23.32	26.56	0.05	-0.15	0.26	0.73	0.57	0.83	0.73	0.57	0.84
Circular movements (s)	53.61	41.19	54.03	40.07	-0.01	-0.18	0.16	0.81	0.69	0.89	0.76	0.62	0.86
Inner surfaces													
Horizontal movements (s)	10.90	12.53	15.64	20.92	-0.23	-0.40	-0.05	0.80	0.68	0.88	0.87	0.77	0.92
Vertical movements (s)	14.58	22.39	15.37	20.36	-0.04	-0.16	0.09	0.89	0.82	0.94	0.90	0.82	0.94

T1: first brushing session; T2: second brushing session; tct: tooth contact time; s: seconds; d: effect size Cohen’s d for differences of means; CI 95%: 95% confidence interval; *all p < 0.001

**Fig 1:**
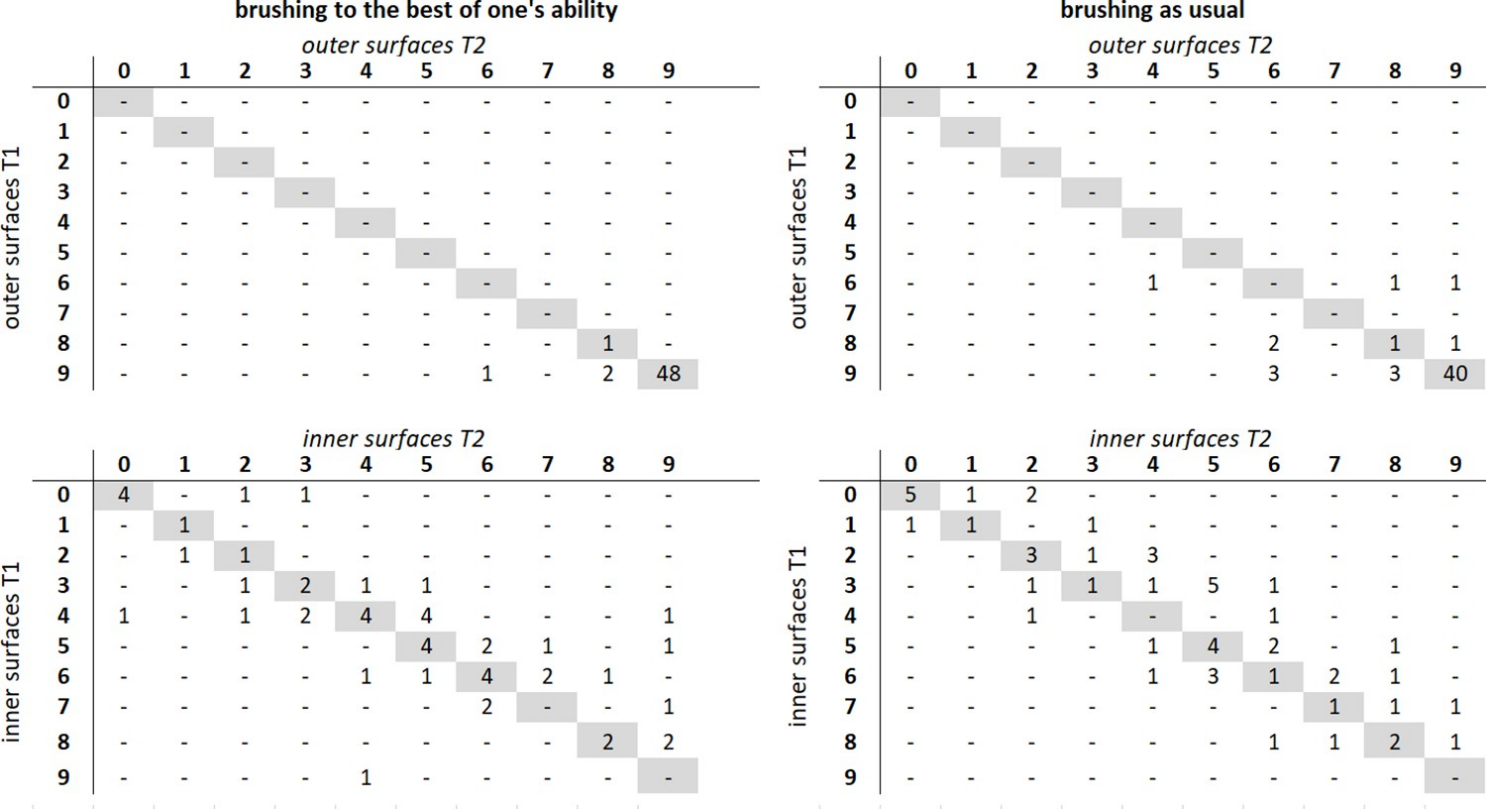
Relationship of QIT-S score between T1 and T2. Cross-tables on the left show the QIT-S values for brushing to the best of one’s ability for the outer and inner surfaces respectively; cross-tables on the right show the QIT-S scores for brushing as usual for the outer and inner teeth surfaces, respectively. QIT-S scores: 0–5, 0–5 sextants were brushed for at least 1 s; 6, each sextant was brushed for at least 1 s but less than 3.5 s; 7 and 8, each sextant was brushed for 3.5–5 s and 5–7.5 s, respectively; 9, all sextants were brushed for at least 7.5 s.

The results of the QIT-S are shown in Fig 1. Significant relationships were found between T1 and T2 in the best brusher group for both the outer surfaces (chi^2^ = 16.65, p < 0.001) and the inner surfaces (chi^2^ = 146.92, p < 0.001). Identical QIT-S values at T1 and T2 were found for the outer surfaces in 94% of individuals and for the inner surfaces in 42% of individuals. Minimal differences (one score point) still indicating similar QIT-S values were found in the outer and inner surfaces of approximately 4% and 35% of the participants, respectively.

In the as-usual brushing group, the relationship between T1 and T2 was also statistically significant (outer surfaces: chi^2^ = 30.23, p < 0.001; inner surfaces: chi^2^ = 114.01, p = 0.001). Identical QIT-S values at T1 and T2 were found in 76% and 34% of individuals for the outer and inner surfaces, respectively. Differences of one score point between T1 and T2 were found in 7.5% of individuals in this group for the outer surfaces and in 30% of individuals for the inner surfaces.

## Discussion

Previous studies observing toothbrushing behavior have been of a cross-sectional nature; therefore, the aim of the present study was to examine whether various aspects of this behavior were stable over time. The study participants brushed their teeth twice at two brushing sessions two weeks apart. One-half of the students brushed their teeth to the best of their ability, while the other half brushed as usual. As toothbrushing behavior is considered to be a routine and automated behavior [26, 28], it was expected that the performance of toothbrushing would be similar at both time points T1 and T2 (i.e. that a person’s position in the group would remain stable over time).

The analyses of the effect sizes of the comparisons between T1 and T2 showed that the average behavior at T2 differed only slightly from that at T1. A small increase in brushing time was observed in both groups (Tables 2 and 3), which may have been a carry-over effect from the procedures of the first brushing session. At T1, plaque measurement was simulated by (sham-) staining after brushing. Although this sham procedure did not give the subjects any realistic feedback about plaque on their teeth, it may have encouraged them to make a greater effort to clean their teeth at T2, thereby increasing their brushing time. However, the differences revealed were small (≤d = 0.23).

Considering the correlations between the observed toothbrushing parameters, a high concordance of the brushing behavior was observed over time. The null hypothesis (H_0_: ρ_BV1,MT_ ≤ 0.5) was rejected for all parameters. In particular, the observed absolute time values were strongly correlated, indicating a stable behavior. This correlation was most pronounced in the total brushing time results for both groups (i.e., tooth contact time).

Similarly, the absolute time values for brushing occlusal and outer and inner surfaces were highly stable over time. In addition, the proportional distribution of time to the tooth surface showed a close relationship between T1 and T2. The proportion of brushing time spent on the respective tooth surfaces was similar to those observed in prior studies on students or adults, with only minor variations [16, 17, 19]. These results suggest that the way individuals brush their teeth seems to be a rather stable behavior which is even more evident when considering the brushing technique (i.e., brushing movements used by the study participants). It was already reported, that among different aspects of toothbrushing the applied brushing technique seems to be the one with the most pronounced habit when repeatedly observed [32]. In previous observations the majority of individuals were shown to apply circular and horizontal movements on the outer tooth surfaces and vertical and horizontal movements on the inner surfaces [15, 17, 21, 22, 34]. Therefore, the present analyses focused on these brushing movements. Although somewhat less pronounced than the other brushing parameters, a clear relationship was found between T1 and T2.

Finally, the results for the QIT-S showed a concordance between T1 and T2. These results differed slightly depending on whether the outer or inner surfaces were considered and whether the study participants brushed their teeth to the best of their ability or as usual. Strong relationships were found with respect to the outer surfaces for both groups. Although slightly less pronounced in the as-usual brushing group, the alterations of the QIT-S shown by some participants were small. The results of the QIT-S for inner surfaces were somewhat different, regardless of how the participants brushed their teeth. In contrast to the outer surfaces, the sextants on the inner surfaces were brushed for less time and some participants did not brush the inner surfaces at all. This neglect of the inner surfaces was observed in several observational studies [8, 15, 17, 20, 21, 34, 39, 40] and could explain the variation observed in this aspect of the brushing behavior. A strong behavioral habit for brushing inner surfaces appears not to be established. Nevertheless, most of the participants showed values scattered around the diagonal, representing complete concordance.

In summary, the data from the present study showed that the toothbrushing behavior exhibited during the two brushing sessions appears to be stable. This corroborates the habit assumption of toothbrushing behavior [26, 28], which was expected since most people perform this behavior regularly daily from an early age. Over time, it becomes a routine behavior that can be performed largely automatically. To date, research on habit formation in the context of toothbrushing behavior focused on aspects such as when or if this behavior occurs [26]. In contrast, how individuals perform this behavior and whether this performance represents a habitual pattern has received little attention. The results of the present study show that toothbrushing behavior appears to be generally routine with little variation. These results are consistent with those of intra-individual toothbrushing patterns observed in a cross-over study, where students brushed their teeth with a manual and a powered toothbrush [20]. In that study, there was no time interval between repeatedly observed toothbrushing; however, when brushing with a different brushing device, study participants tended to show similar behavioral patterns [20].

The development of a stable individual pattern of toothbrushing performance implies that this behavior encompasses both strengths and deficits. With regard to the deficits, such a stable behavior is much harder to change than a brushing behavior that would only be dysfunctional by chance. The results indicate that increasing the frequency of toothbrushing is likely insufficient to improve oral hygiene and that preventive measures should consider the difficulty of changing established habits. Healthcare professionals and patients must recognize this challenge when addressing oral hygiene deficiencies.

The present study has both strengths and limitations that should be discussed. One of the strengths is the systematic analysis of various aspects of toothbrushing behavior, such as total brushing time, distribution on tooth surfaces and sextants, and brushing technique, which provides a more thorough understanding of the behavior than assessing only one or a few factors. However, other important aspects of toothbrushing behavior have not been addressed here. These include the brushing force or the sequence of brush positions and the number of times brushing areas are changed. However, previous reports have shown that these aspects are similar among different brushing sessions [32, 33]. The use of thoroughly trained and calibrated video observers and the high standardization in the performance of the study are strengths to be highlighted. The study procedures at T1 and T2 were kept as constant as possible to ensure that toothbrushing was performed under the same conditions by each participant. To avoid visual feedback of remaining plaque at T1, which could result in a change in brushing behavior, a sham staining procedure was performed at T1. However, it cannot be excluded that this sham procedure caused a change in the behavior, and it is possible that it was responsible for the longer brushing times at T2 compared to T1. Another limitation was the external validity of the results. First, the present study analysed university students only and the generalizability to other age groups is undetermined. However, toothbrushing routines are acquired through regular and frequent practice; therefore, older persons could show an even more pronounced habit. Indeed, it would be interesting to study children in this regard. Secondly, the stability of this behavior was demonstrated under laboratory conditions and it is unclear whether individuals exhibit the same level of stability when brushing their teeth outside of this environment. Further studies analyzing toothbrushing in a domestic setting would be instructive.

## Conclusion

The findings showed that repeatedly assessed toothbrushing performance exhibited a high degree of stability. The observed total brushing time and distribution of time to tooth surfaces and brushing movements were highly correlated. Deficits in brushing behavior such as neglecting the inner tooth surfaces and omitting entire sextants were similarly evident at both time points of observation.

## Supporting information

S1 File Supplemental material. (PDF)
