# Supplementary material for: Toothbrushing behavior over time: A correlational analysis of repeatedly assessed brushing performance
Source: PLoS One. 2024 Dec 19;19(12):e0296724. doi: 10.1371/journal.pone.0296724 (PMC11658570; doi:10.1371/journal.pone.0296724)
Supplement: S1 File — (PDF) [file pone.0296724.s001.pdf]

Supplemental Material

Table of content

1.Procedure 2

2.Assessement of oral health 3

3.Scatterplots of behavioral parameters (T1 / T2).. 4

1. Procedure at the second brushing session (T2) [cited verbatim from Weik et al., 2023; BMC Oral Health 23:456. https://doi.org/10.1186/s12903-023-03127-3]

“Procedure:
Students interested in study participation were contacted by telephone to provide detailed information about the study, and the inclusion/exclusion criteria were checked. Eligible students were scheduled for two appointments that were two weeks apart. While study participants were asked to brush their teeth at both appointments, plaque after brushing was only assessed at the second appointment. The present study therefore focuses on the data assessed at the second appointment.
All participants were instructed to refrain from any oral hygiene behavior at least four hours before the appointments. Upon arrival at the laboratory rooms of the Institute of Medical Psychology, Justus-Liebig-University of Giessen, an assistant (A1) who was neither involved in the assessment of dental parameters nor the video recording while brushing welcomed the students and led them into the dental examination rooms. Dental plaque was assessed by one of the two dentists (TS or D2). Each dentist performed plaque assessments in 50% of the study participants. Afterward, A1 led the study participants to another room for tooth brushing where another assistant (A2) welcomed the participants. A2 accompanied them into an adjacent room equipped with a washbasin and a tablet computer with a front camera fixed at a tripod in front of the participants. This front camera served both as a mirror and as a recording tool for video recording of the participants’ tooth brushing performance. A red transparent sheet covered the surface of the tablet display to make plaque staining invisible for the participant. There were two side cameras at the walls for additional recordings used in case the tablet camera did not fully capture the brushing event. The participants were provided with a standard manual toothbrush (Elmex InterX short brush-head, medium; GABA, Loerrach, Germany) and toothpaste (Elmex; GABA, Loerrach, Germany). Additionally, dental floss (waxed and unwaxed dental floss; Elmex; GABA, Loerrach, Germany), super floss (Meridol Special-Floss; GABA, Loerrach, Germany) and interdental brushes (Elmex interdental brush sizes 2 and 4; GABA, Loerrach, Germany) were provided on a table beneath the basin. A2 informed the participants that these devices were at their free disposal. He then gave them the brushing instruction corresponding to their experimental condition (see below). Afterward, he asked them not to start brushing until they were told to do so over an intercom system. He then went to the adjacent room from which he started the video recording and repeated the respective instruction via intercom and asked them to start with tooth brushing. Participants communicated via intercom when they had finished their brushing. Immediately afterward, A2 led them back to the dental examination room where plaque was assessed again. At the end of the examination, participants were led to a neutral examination room and completed the questionnaire assessing their self-perceived oral cleanliness (SPOC) [22] as well as other questionnaires which were not within the scope of the present study. All questionnaires were presented online via a tablet computer using SoSci Survey [23] and made available to study participants at www.soscisurvey.de.”

2. Assessment of oral health [cited verbatim from Weik et al., 2023; BMC Oral Health 23:456. https://doi.org/10.1186/s12903-023-03127-3]

“Oral health status
For clinical description of the study groups, dental status (decayed, missing and filled teeth), the Papillary Bleeding Index (PB [27] modified by Rateitschak [28]) and the periodontal screening index (PSI; [29]) were assessed prior to tooth brushing. PBI was determined at the outer and inner surfaces. Scores range from 0 to 4: 0, no bleeding on probing; 1, single bleeding point(s); 2, several bleeding points or thin line; 3, interdental triangle filled with blood; 4, profuse bleeding on probing”.

Dental status was assessed at the first brushing session (T1) prior to toothbrushing. The PBI was assessed at both brushing sessions (T1 and T2) prior to toothbrushing.

3. Scatterplots of behavioral parameters (T1 and T2)

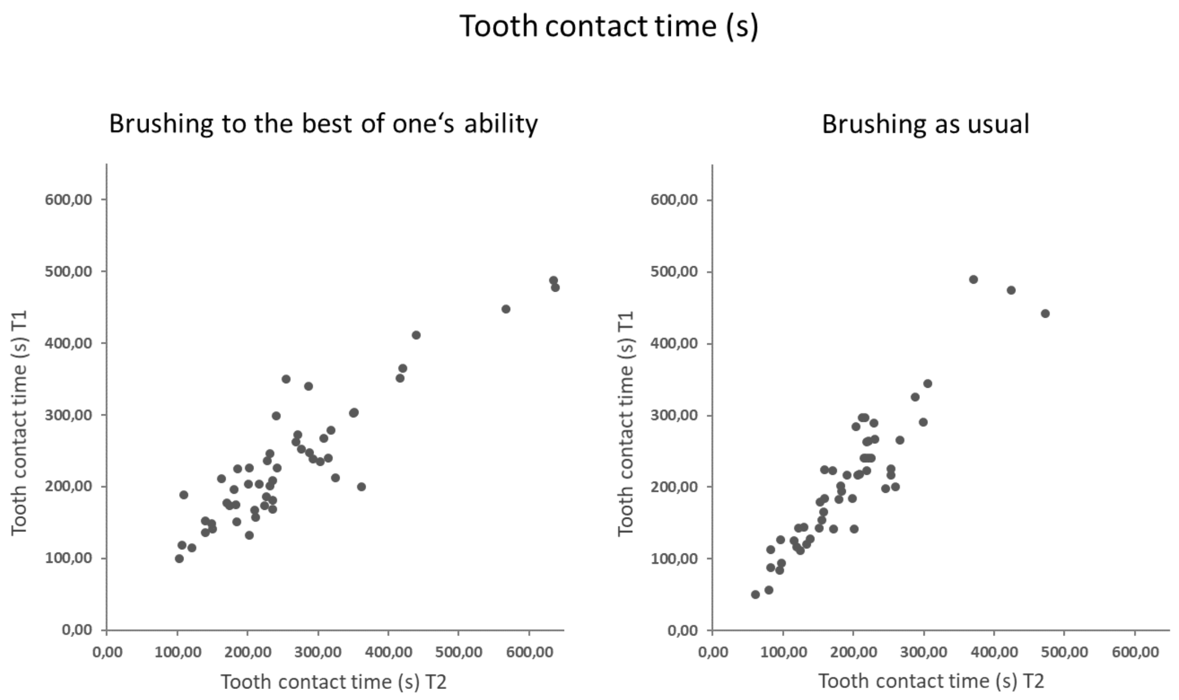

Tooth contact time (seconds) assessed at the first brushing session (T1) and the second brushing session (T2) for study participants brushing to the best of their ability (left scattlerplot) and brushing as usual (right scatterplot).

Occlusal surfaces (s)

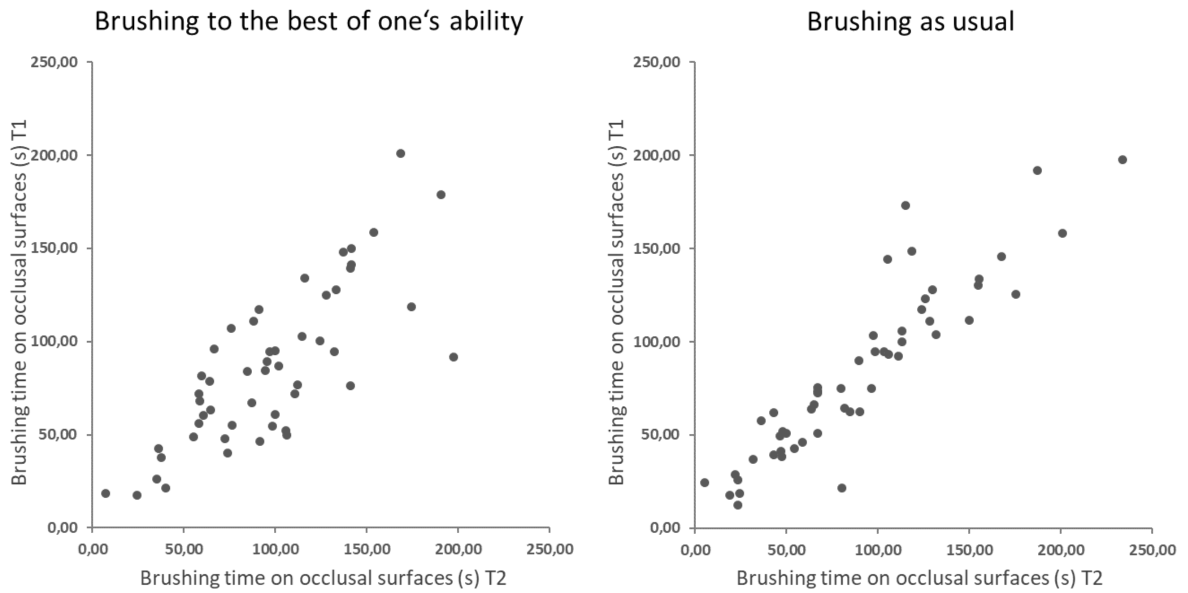

Time (seconds) brushing the occlusal surfaces assessed at the first brushing session (T1) and the second brushing session (T2) for study participants brushing to the best of their ability (left scattlerplot) and brushing as usual (right scatterplot).

Occlusal surfaces (% tct)

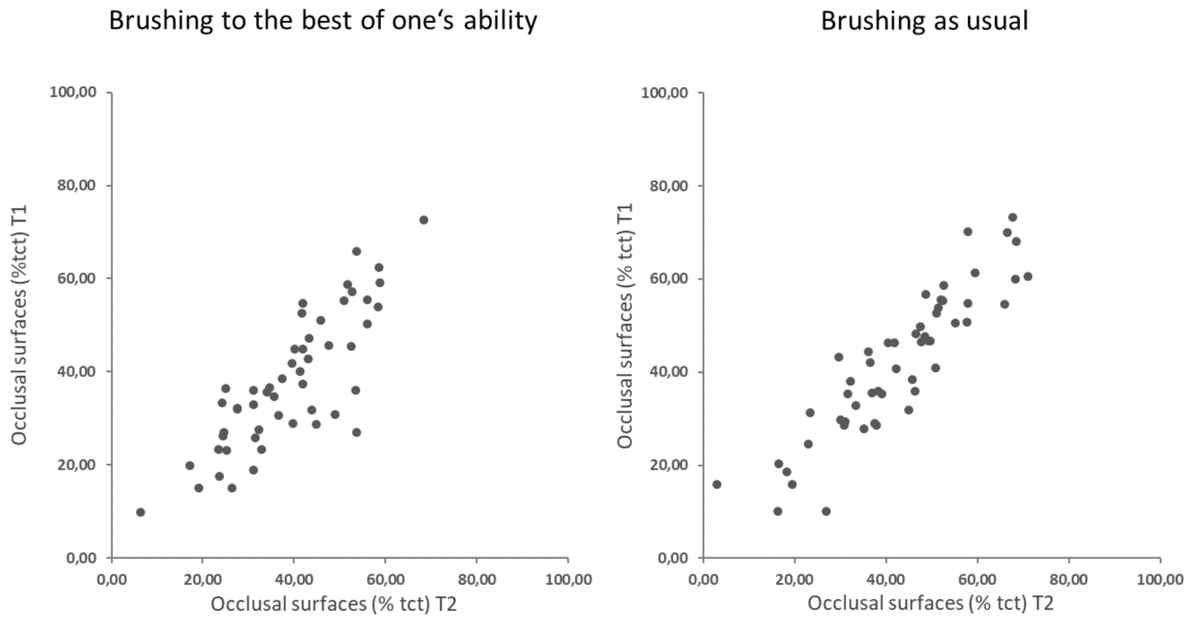

Proportion of tooth contact time spent by brushing the occlusal tooth surfaces assessed at the first brushing session (T1) and the second brushing session (T2) for study participants brushing to the best of their ability (left scattlerplot) and brushing as usual (right scatterplot).

Outer surfaces (s)

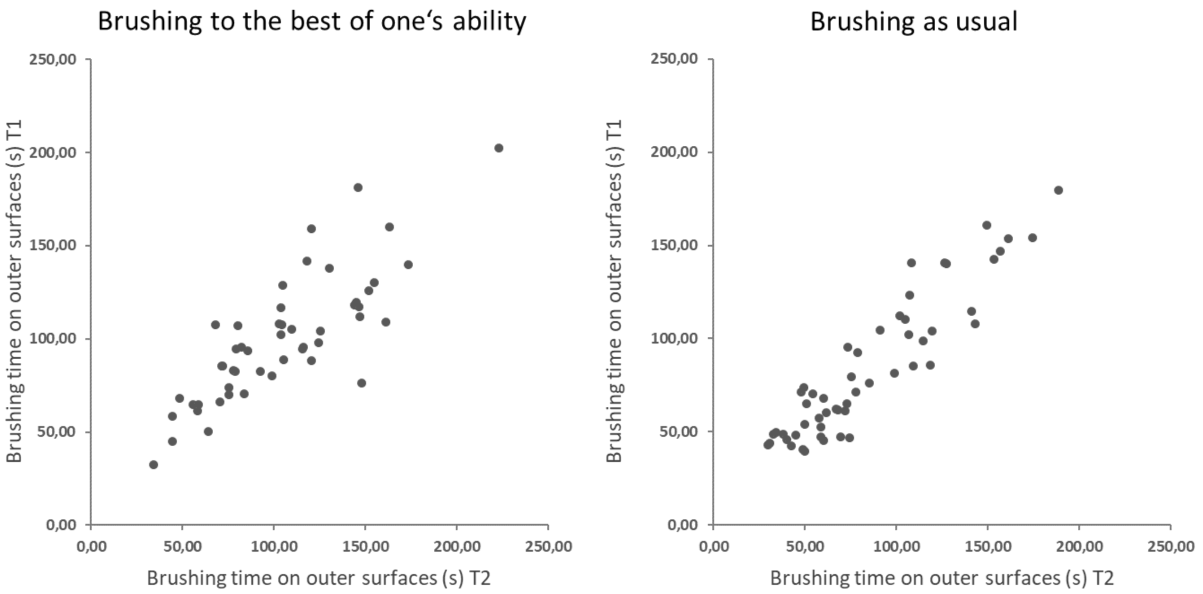

Time (seconds) brushing outer surfaces assessed at the first brushing session (T1) and the second brushing session (T2) for study participants brushing to the best of their ability (left scattlerplot) and brushing as usual (right scatterplot).

Outer surfaces (% tct)

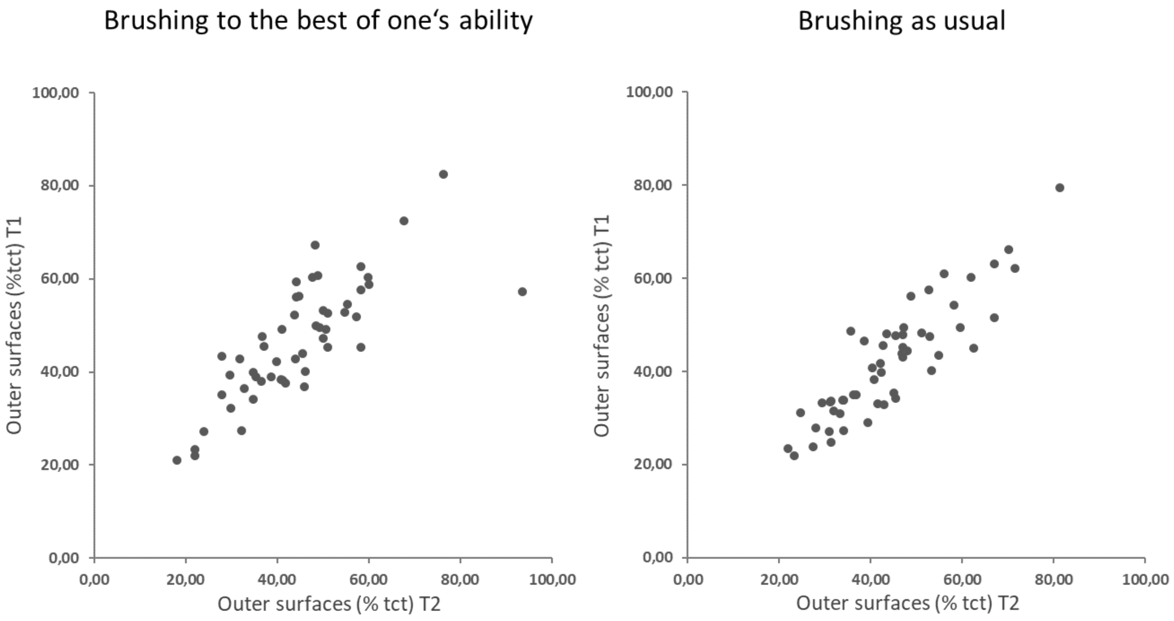

Proportion of tooth contact time spent by brushing the outer tooth surfaces assessed at the first brushing session (T1) and the second brushing session (T2) for study participants brushing to the best of their ability (left scattlerplot) and brushing as usual (right scatterplot).

Inner surfaces (s)

Brushing to the best of one‘s ability

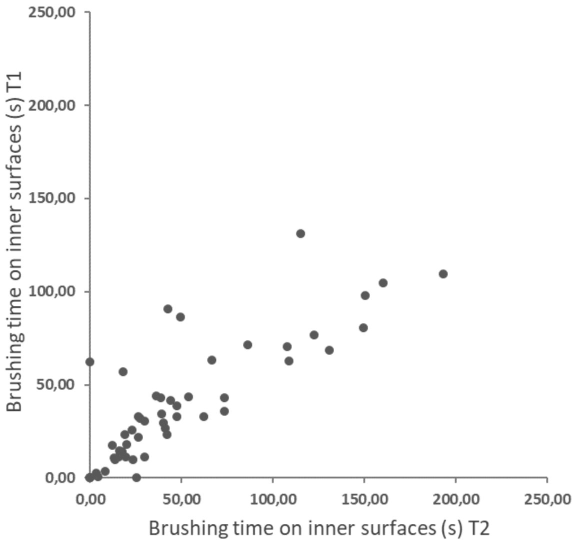

Brushing as usual

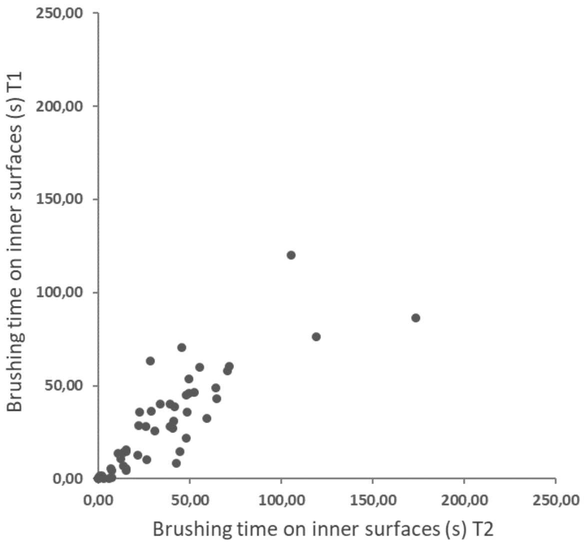

Time (seconds) brushing the inner tooth surfaces assessed at the first brushing session (T1) and the second brushing session (T2) for study participants brushing to the best of their ability (left scattlerplot) and brushing as usual (right scatterplot).

Inner surfaces (% tct)

Brushing to the best of one‘s ability

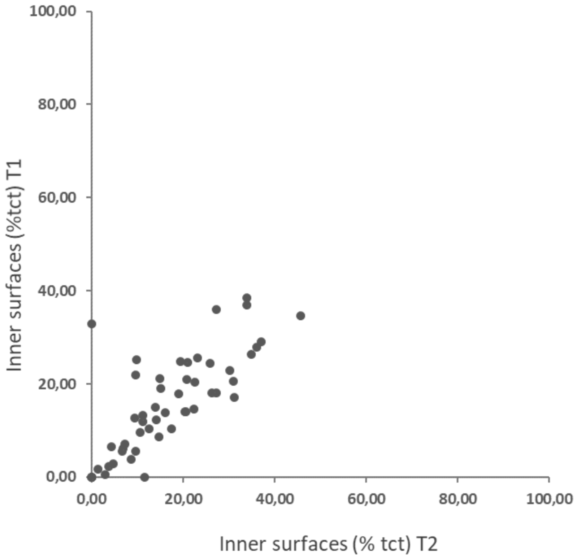

Brushing as usual

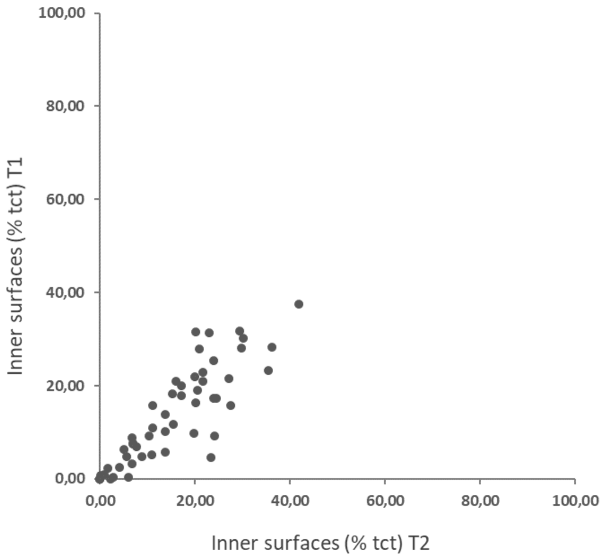

Proportion of tooth contact time spent by brushing the inner tooth surfaces assessed at the first brushing session (T1) and the second brushing session (T2) for study participants brushing to the best of their ability (left scattlerplot) and brushing as usual (right scatterplot).

Horizontal brushing movements at outer surfaces (s)

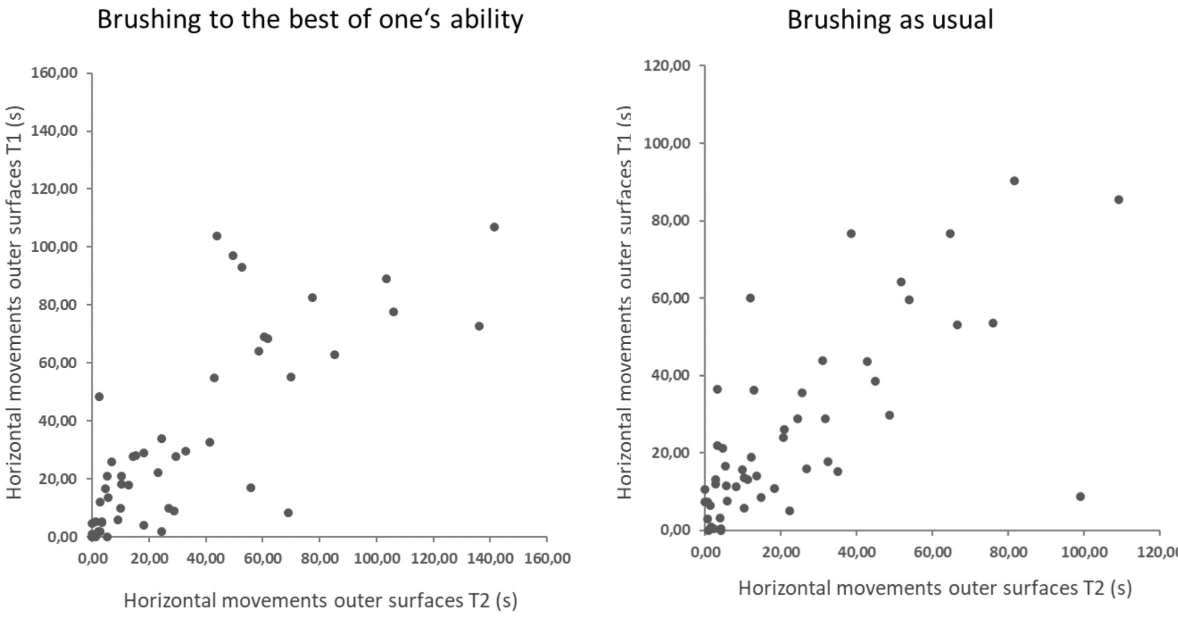

Time (seconds) spent by horizontal brushing movements on the outer tooth surfaces assessed at the first brushing session (T1) and the second brushing session (T2) for study participants brushing to the best of their ability (left scattlerplot) and brushing as usual (right scatterplot).

Circular brushing movements on outer surfaces (s)

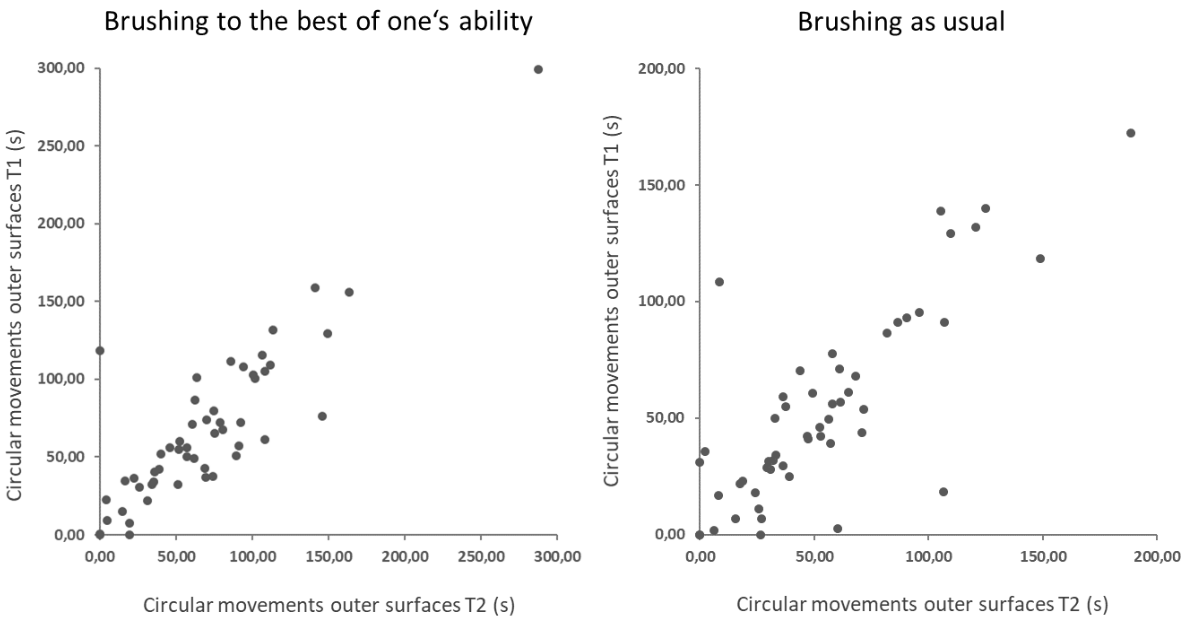

Time (seconds) spent by circular brushing movements on the outer tooth surfaces assessed at the first brushing session (T1) and the second brushing session (T2) for study participants brushing to the best of their ability (left scattlerplot) and brushing as usual (right scatterplot).

Horizontal brushing movements on inner surfaces (s)

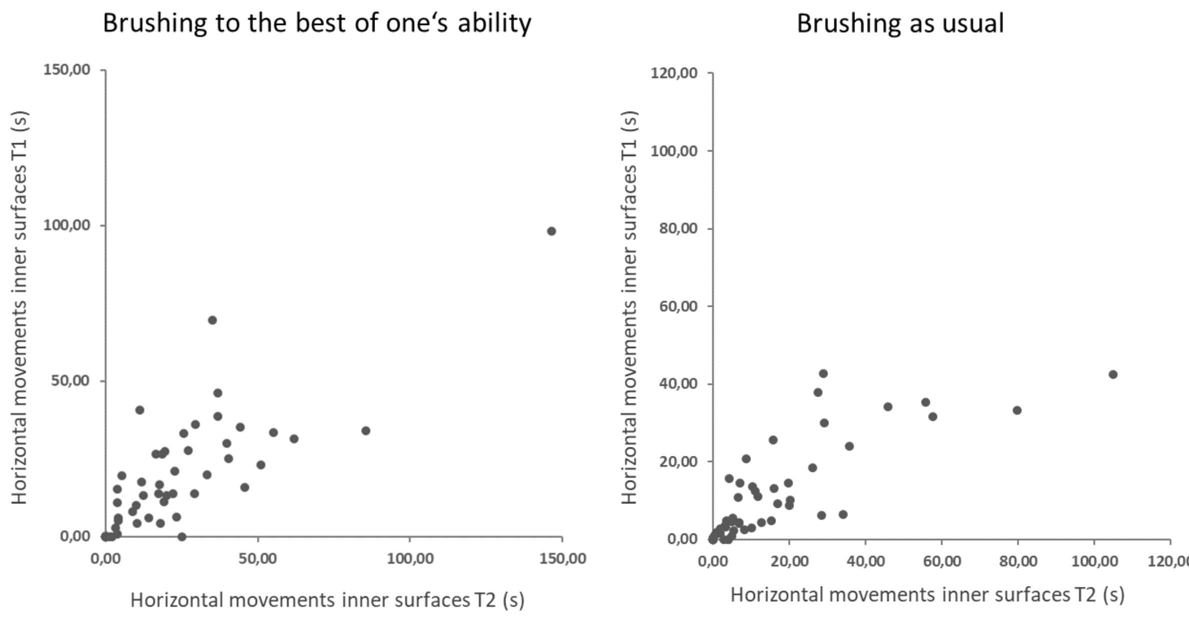

Time (seconds) spent by horizontal brushing movements on the inner tooth surfaces assessed at the first brushing session (T1) and the second brushing session (T2) for study participants brushing to the best of their ability (left scattlerplot) and brushing as usual (right scatterplot).

Vertical brushing movements on inner surfaces (s)

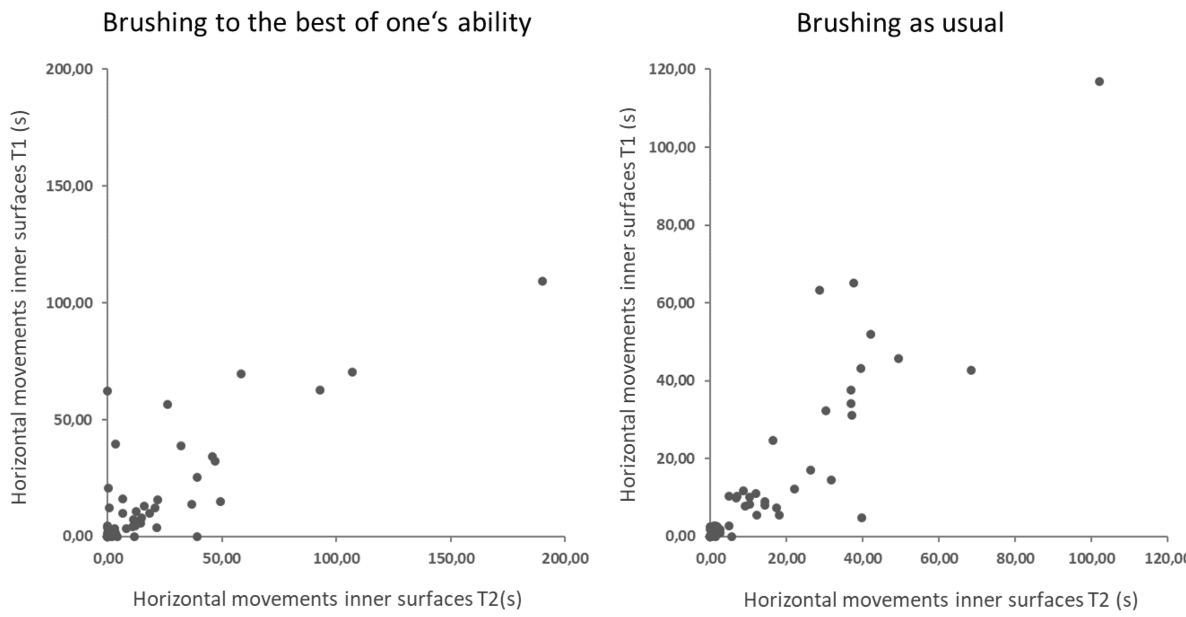

Time (seconds) spent by vertical brushing movements on the inner tooth surfaces assessed at the first brushing session (T1) and the second brushing session (T2) for study participants brushing to the best of their ability (left scattlerplot) and brushing as usual (right scatterplot).
